# Why Do We Need Multifunctional Neuroprotective and Neurorestorative Drugs for Parkinson’s and Alzheimer’s Disorders?**

**DOI:** 10.5041/RMMJ.10011

**Published:** 2010-10-31

**Authors:** Moussa B. H. Youdim

**Affiliations:** Eve Topf and US National Parkinson Foundation Centers of Excellence for Neurodegenerative Diseases, The Bruce Rappaport Faculty of Medicine, Technion - Israel Institute of Technology, Haifa, Israel, and Department of Biology, Yonsei University, Seoul, Korea

**Keywords:** Rasagiline multimodal drugs, antiapoptotic, neuroprotection, neurorestoration, Parkinson’s disease, Alzheimer’s disease

## Abstract

Parkinson’s disease (PD) and Alzheimer’s disease (AD) are severe neurodegenerative disorders, with no drugs that are currently approved to prevent the neuronal cell loss characteristic in brains of patients suffering from PD and AD, and all drug treatments are symptomatic and monomodal in their action. Due to the complex pathophysiology, including a cascade of neurotoxic molecular events that result in neuronal death and predisposition to depression and eventual dementia, and etiology of these disorders, an innovative approach towards neuroprotection or neurorestoration (neurorescue) is the development and use of multifunctional pharmaceuticals which can act at different brain regions and neurons. Such drugs target an array of pathological pathways, each of which is believed to contribute to the cascades that ultimately lead to neuronal cell death. In this short review, we discuss examples of novel multifunctional ligands that may have potential as neuroprotective-neurorestorative therapeutics in PD and AD, some of which are under development. The compounds discussed originate from synthetic chemistry as well as from natural sources.

## INTRODUCTION

Parkinson’s disease (PD) is an age-related neurodegenerative disease with progressive loss of dopaminergic (DA) neurons in the substantia nigra pars compacta (SNpc). In patients, this depletion of neurons presents clinically with severe motor symptoms including uncontrollable resting tremor, bradykinesia, rigidity, and postural imbalance.^1^–^3^ These symptoms, which affect 1% of individuals over the age of 65, start to manifest when 70%–80% of DA neurons in the SNpc are lost.^4^,^5^ The exact etiology of PD remains to be fully elucidated, but the key theories propose either an environmental (e.g. insecticides^6^–^8^) or a genetic (e.g. parkin^9^,^10^) origin, or a combination of both.

In 2009, the market value for PD and AD therapies exceeded US$6.5 billion, with projections that these will surpass cancer as the second most common cause of death of the elderly.^3^ Therefore, there is a real sense of urgency to discover novel therapies for the treatment or, preferably, prevention of these diseases. Currently the only therapies approved for the treatment of PD and AD are agents that attenuate the symptoms (symptomatic) of the disease without disease-modifying activity except the anti-Parkinson drug rasagiline (Rasagiline),^11^ which we developed.^12^ The mainstay for PD treatment focuses on the replacement of lost DA with L-dopa, dopamine agonists, monoamine oxidase B inhibitors, and catechol-O-methyl transferase inhibitors, thereby normalizing the patient symptomatically;^10^ while for AD there are the cholinesterase inhibitors and the glutamate antagonist memantine. Tragically, but importantly in view of the seriousness of disease progression, the fact is that the course of the disease is not affected by the utilization of these drugs, and the loss of neurons continues unabated even as symptoms may be controlled, at least following initial treatment. Currently, no drugs with claimed neuroprotective activity have been approved by the Food and Drug Administration (FDA) for the treatment of PD or AD (Table 1).^5^,^13^ Significantly though, recent research has suggested that some drugs used for symptomatic relief in PD, such as rasagiline, pramipexole,^14^–^16^ and memantine,^17^–^19^ may also possess neuroprotective activities; rasagiline is currently the only drug that may have a disease-modifying activity.

**Table 1:** Definitions of the terms neuroprotection, neurorestoration, and neurorescue.

**Neuroprotection**
A beneficial interaction that prevents or slows neurons from dying.^5^ A need for disease pre-symptomatic biomarker.
**Neurorestoration**
A beneficial interaction that replaces dying or dead neuronal cells with viable cells.^5^ Acting during the symptomatic phase.
**Neurorescue**
A beneficial interaction that rescues cells where neuronal cell death has already started.^17^ Acting during symptomatic phase.

Recent literature shows that there has been a paradigm shift in the way researchers are considering the development and design of drugs to treat diseases with complex etiological pathways (i.e. diseases with multiple drug targets).^20^–^27^ In a pathway system with a multitude of drug targets, a drug with a single-target mechanism of action cannot always compensate for or correct a complex pathway, which suggests that a complex pathway disease should be treated 1) with a multitude of molecules, each acting on different pathways in the disease (polypharmacy), or 2) with one molecule that possesses promiscuous activity acting on different pathways (multiple mechanism drugs).

**Figure 1:**
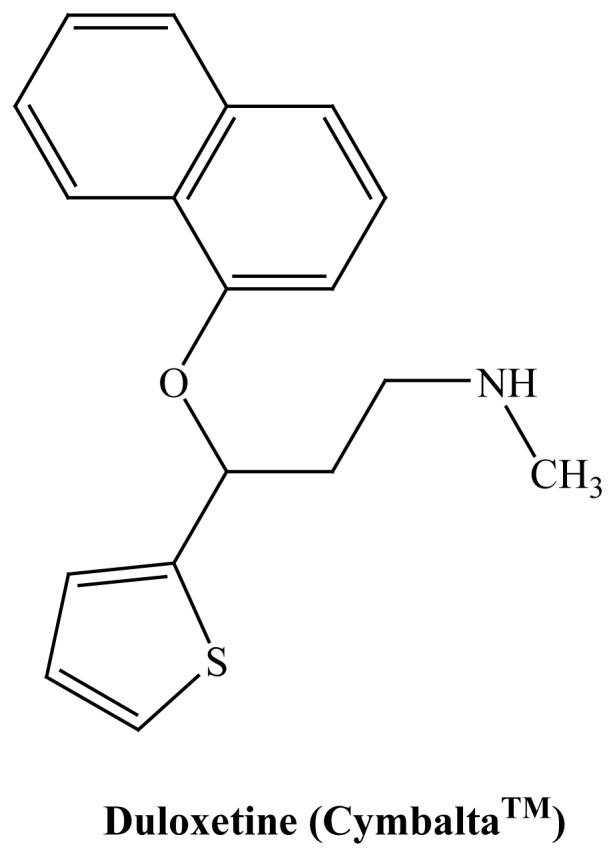
Structure of a multimodal antidepressant that acts both as serotonin and norepinephrine uptake inhibitor.

Polypharmacy, therefore, is the clinical practice of combining two or more medications in a patient’s medication profile, with a view to treat one specific disease. For example, the combination use of salmeterol (a β2-adrenergic agonist) and fluticasone (a glucocorticoid steroid) in asthma has led to the combination of these two medications in one (Advair®). Also, the combination (in Vytorin®) of simvastatin [an 3-hydroxy-3-methyl-glutaryl-coenzyme A (HMG-CoA) reductase inhibitor] and ezetimibe (an inhibitor of dietary cholesterol uptake) is used to treat hyperlipidemia.^20^ The major dilemma encountered in a polypharmaceutical approach is a significant chance of increases in side-effects, which may be reduced statistically with the use of only one compound. The recent appearance on the market of drugs that display two mechanisms to treat a particular disease has been a clear move in the direction of the latter paradigm. One example, duloxetine (Cymbalta®) (Figure 1), used in the treatment of depression, inhibits both serotonin and norepinephrine uptake in the central nervous system (CNS).^28^–^30^ The introduction of drugs such as duloxetine indicates the clinical feasibility of designing multifunctional ligands to treat CNS disorders with complex disease pathways.

**Figure 2:**
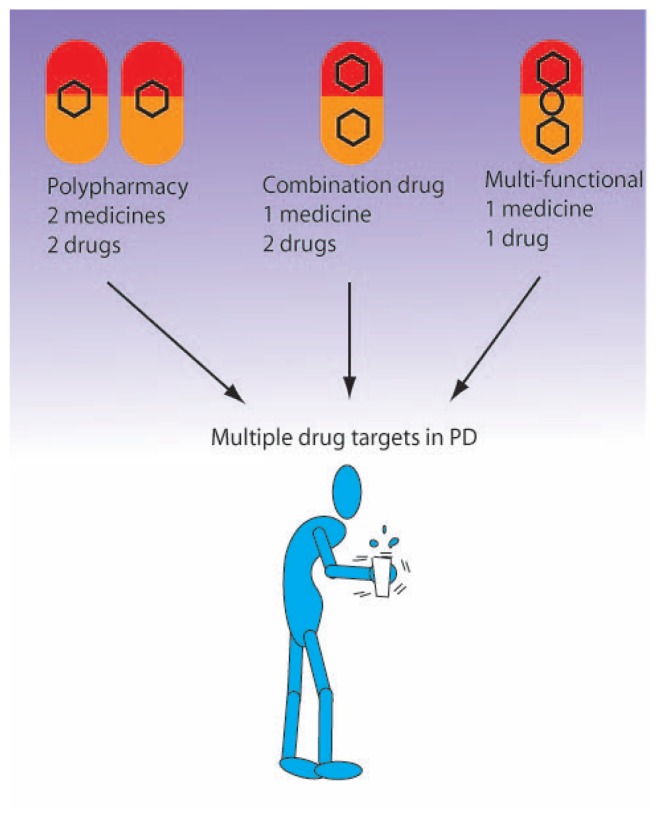
Three approaches towards combination drug therapy in a multi-target disease. These include: 1) giving a combination of two or three drugs, i.e. separate medicines, 2) chemically combining two drugs into one medicine, and/or 3) synthesizing one drug possessing several pharmacologically active moieties that can act on specific enzymes and/or receptors.

In this review, we will consider examples of compounds with multifunctional neuroprotective-neurorescue (Figure 2 and see Table 1 for definitions) properties that may have promise in the treatment of PD, and similar approaches have been made for multimodal drugs for AD,^31^,^32^ but for the present discussion we shall focus mainly on PD. Some of the compounds discussed were discovered through serendipity, while others were the products of active drug design projects.

## RASAGILINE

**Figure 3:**
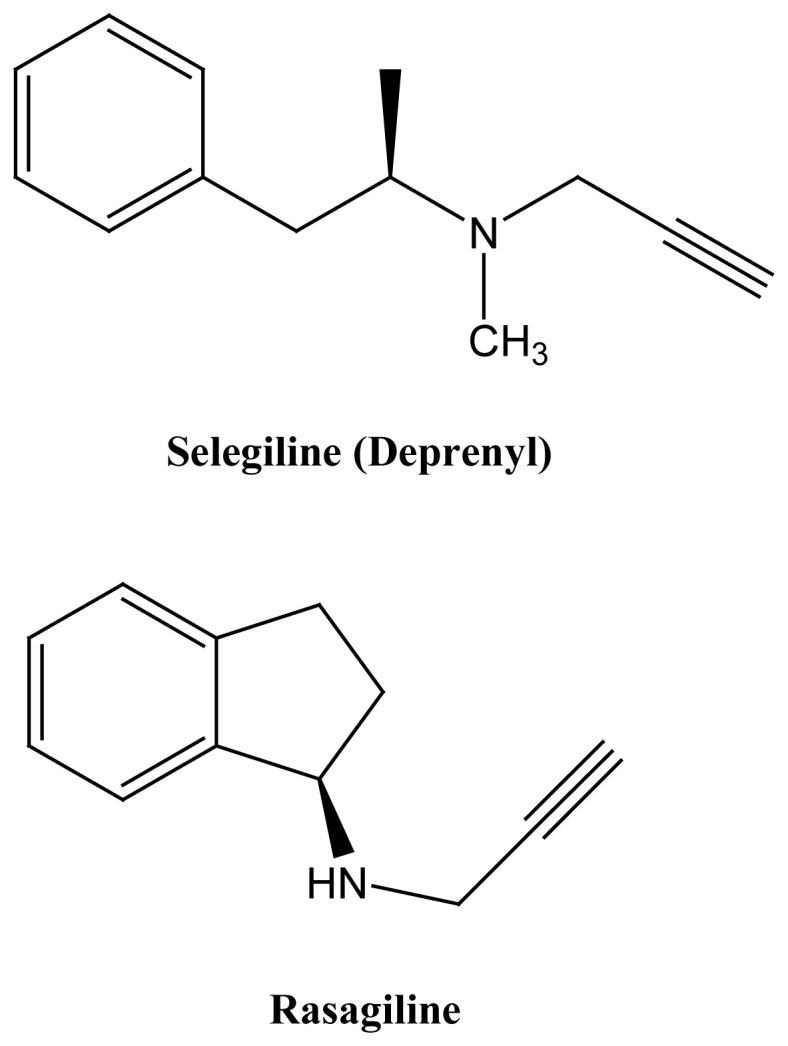
Structures of the anti-Parkinson MAO-B inhibitors selegiline and rasagiline as monomodal drugs.

Rasagiline is a restricted analog of selegiline (Figure 3) and is a newly approved compound for the treatment of PD.^10^ Rasagiline (*N*-propargyl-1*R*-aminoindan) is an anti-PD drug with selective MAO-B-inhibitory activity.^33^ Its *S*-isomer, TVP1022 (*N*-propargyl-1*S*-aminoindan), is more than a 1,000 times less potent as an MAO inhibitor than rasagiline but still retains neuroprotective activity, which suggests that the propargylamine moiety (even when ostensibly not involved in Michael reaction chemistry at the flavin adenine nucleotide (FAD) co-factor within the MAO catalytic site as the processing group in suicide inhibition) is responsible for the neuroprotective activity seen in both these compounds.^34^–^37^

The selectivity of rasagiline as an MAO-B inhibitor compared with TVP1022 is thought to be associated with the ability of rasagiline to enter the catalytic site gorge of MAO-B. On the other hand, the configuration of the *S*-isomer imparts a highly restrictive conformation on the enzyme–ligand complex, which prevents the molecule from entering the catalytic site, precluding it from acting as a mechanism-based inhibitor. Interestingly, the neuroprotective activity associated with these compounds has now been shown to be associated with the ability of propargylamine^36^,^37^ to protect mitochondrial viability by activation of Bcl-2 and protein kinase C (PKC)-α and -ɛ and by down-regulating proapoptotic FAS and Bax, and PKCδ and -γ.^33^ Additionally, these drugs induce the release of the soluble neuroprotective-neurotrophic form of the amyloid precursor protein α (sAPPα) through a PCK-MAP-mediated activation of α-secretase.^27^

**Figure 4:**
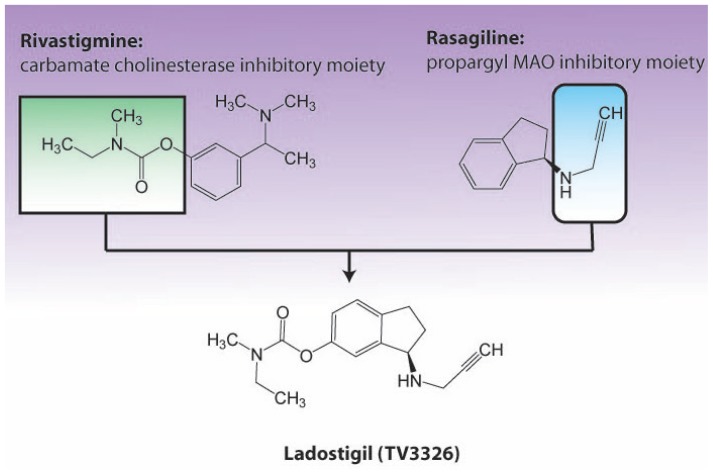
Design of the multimodal anti-Alzheimer drug ladostigil, where the carbamate cholinesterase inhibitor moiety is introduced into rasagiline in order also to posses the monoamine oxidase inhibitory and neuroprotective activity of the latter drug.

The identification of the propargylamine moiety as a key element that confers neuroprotective activity and, in cases such as rasagiline and selegiline, also MAO-inhibitory activity, led to the development of acetylcholinesterase (AChE) inhibitors such as ladostigil (TV3326, now in phase II clinical studies), another anti-Alzheimer’s disease/anti-Parkinson’s/ antidepressant drug. 27,33,38,39 Ladostigil (Figure 4) is a dual acetylcholine-butyrylcholine-esterase and brain-selective MAO-A/B inhibitor *in vivo*, designed by combining the carbamate cholinesterase inhibitory moiety found in the rivastigmine molecule, with the pharmacophore of rasagiline and TVP1022, both of which possess the propargylamine moiety. Ladostigil has been shown to have antidepressant activity, due to its ability to inhibit MAO-A in the raphe nucleus, striatum, hippocampus, and hypothalamus, and to raise brain levels of DA, norepinephrine, and serotonin.^39^ Its ability also to inhibit MAO-B attenuates 1-methyl-4-phenyl-1,2,3,6-tetrahydropyridine (MPTP) toxicity in mice, a rodent model of Parkinsonism.^40^ Although a poor MAO-B inhibitor, the *S*-isomer of ladostigil, TV3279, has shown similar neuroprotective activity to rasagiline and ladostigil *in vitro* and in laboratory animals,^27^ with molecular mechanisms apparently identical to that of rasagiline.

## IRON CHELATORS WITH RADICAL SCAVENGING AND BRAIN-SELECTIVE MONOAMINE OXIDASE-INHIBITORY ACTIVITY

Degenerating nigrostriatal DA neurons are the main pathological feature in the SNpc of PD sufferers. In addition, many PD patients also experience dementia and depression that likely result from sporadic neurodegeneration in cholinergic, noradrenergic, and serotonergic pathways. In PD, accumulation of iron is found inside some melanin-containing DA neurons and inside amyloid plaques and neurofibrillary tangles associated with PD dementia.^41^ It has been suggested that iron accumulation may contribute to the oxidative stress-induced apoptosis reported in both PD and PD dementia.^34^,^41^ Such oxidative stress may result from increased glial MAO activity leading to exacerbated hydrogen peroxide production that can generate reactive hydroxyl radicals through Fenton chemistry with intracellular ferrous iron. Iron chelators such as desferoxamine, clioquinol, and VK-28 have been shown to have neuroprotective activity in animal models of AD and PD.^41^

**Figure 5:**
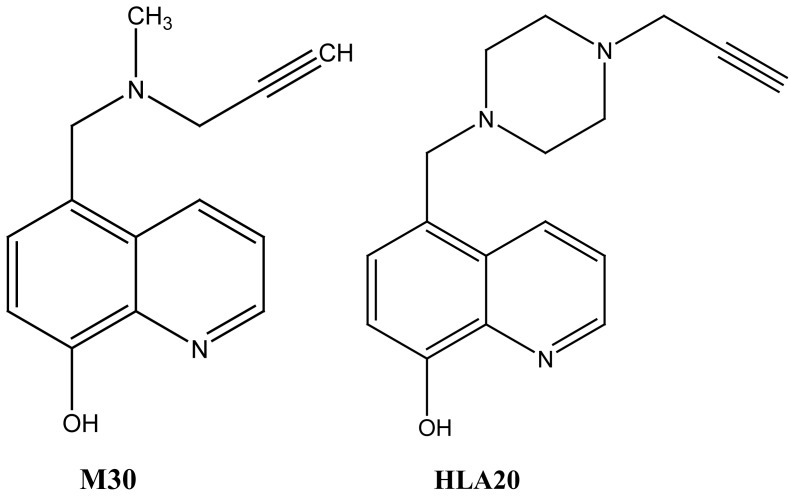
Structures of multimodal anti-Parkinson/anti-Alzheimer drugs derived from the iron chelator VK-28. These compounds possess iron-chelating, radical-scavenging plus neuroprotective activity of rasagiline.

**Figure 6:**
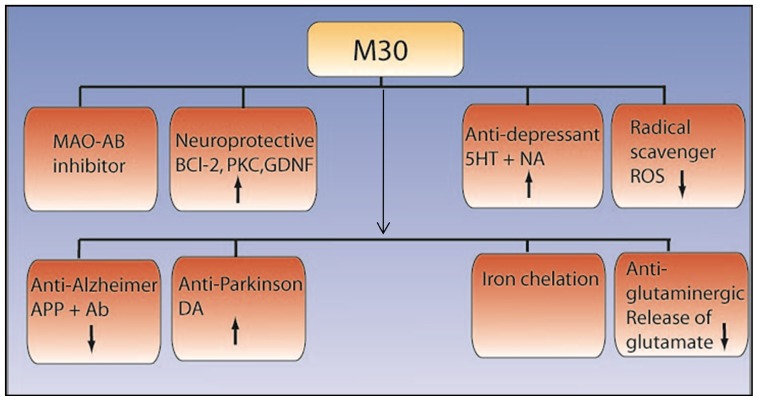
Neuroprotective anti-Alzheimer, anti-Parkinson, and antidepressant effects of M30. See text for discussion. ROS, radical oxygen species, NA, noradrenaline.

Based on this proposal, Zheng et al.^42^ developed neuroprotective compounds with dual iron-chelating and MAO-B-inhibitory activity. These authors combined the antioxidant chelator moiety present in an 8-hydroxyquinoline derivative of the neuroprotective brain-permeable iron chelator VK-28, with the propargylamine moiety (found in compounds such as rasagiline and selegiline, as stated earlier). HLA20 was identified as a potential lead compound for further studies, having selectivity for MAO-B with an IC_50_ value in the region of 110 μM (>200 μM for MAO-A), as well as acting as a free radical scavenger. However, a related compound designated M30 [5-(N-methyl-N-propargylaminomethyl-8-hyd-roxyquinoline], unlike HLA20 (Figure 5) was found, *in vitro*, to be a highly potent MAO-A and B inhibitor, with brain selectivity for these enzymes *in vivo*, in addition to possessing iron-chelating properties similar to desferoxamine.^23^,^35^,^42^ M30 (Figure 5, Figure 6) behaves similarly to other propargylamine MAO inhibitors by acting as a suicide- or mechanism-based inhibitor after being identified and processed as a substrate by the enzyme and imparts similar neuroprotective properties as those found in rasagiline and ladostigil. M30 protects against MPTP and kainate neurotoxicity in mice by virtue of both its MAO-inhibitory and iron-chelating–radical-scavenging properties in these two animal models of neurodegeneration. M30 has recently been shown to have dopaminergic neurorestorative activity *post* treatment with MPTP^43^ and lactacystin^44^ in models of PD. The neurogenic activity of M30 and HLA20 has been attributed to the inhibition of iron-dependent prolyl-4-hydroxylase, via chelation of iron resulting in activation of hypoxia-inducing factor (HIF) that regulates transcription of a series of neurotrophins such as brain-derived neurotrophic factor (BDNF), glial cell line-derived neurotrophic factor (GDNF), erythropoietin, and vascular endothelial growth factor (VEGF). The consequence of HIF activation is inhibition of cell cycle G^0^/G^1^, resulting in inhibition of cyclin D1 that causes cell arrest differentiation into neurons as seen in the neurorestorative activity of M30 in the two models of PD.^43^–^45^

**Figure 7:**
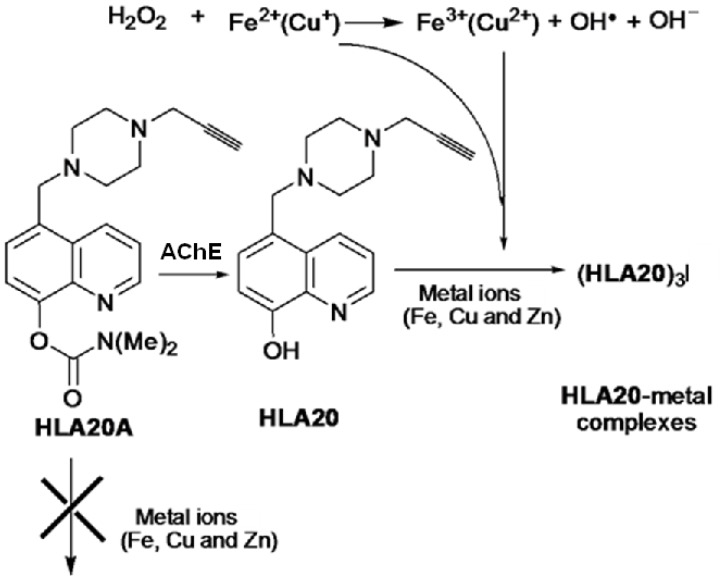
Novel multimodal cholinesterase–iron-chelating–radical-scavenging drug, HLA20A, for Alzheimer’s disease derived from HLA20. The drug acts by causing pseudo-inhibition of cholinesterase and releasing HLA2.

**Figure 8:**
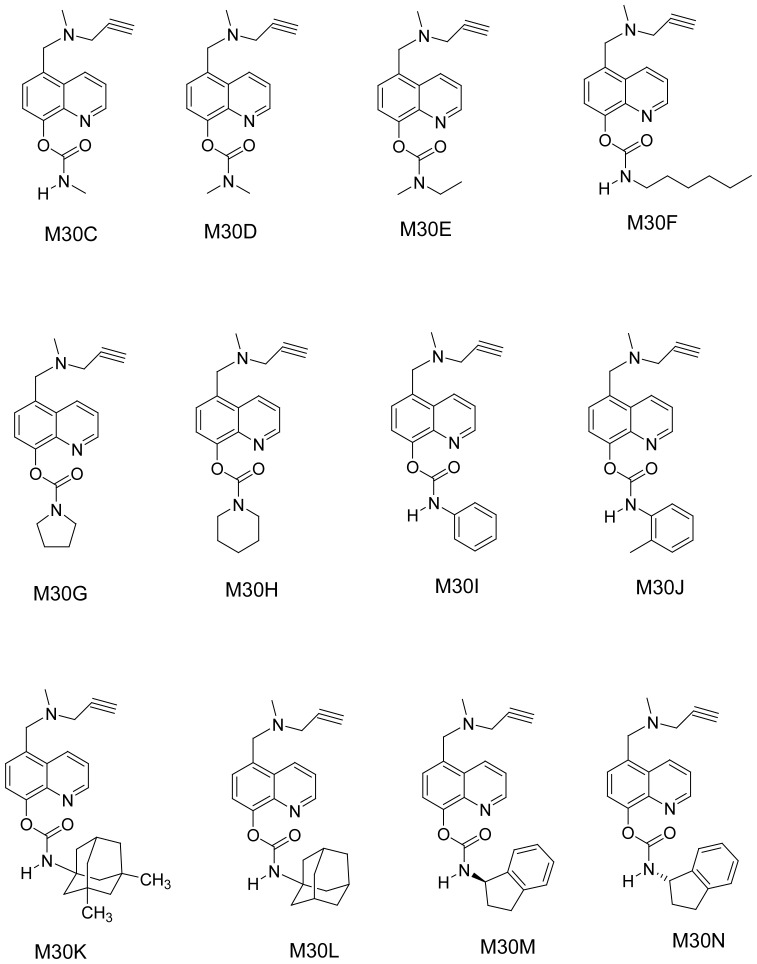
Novel multimodal cholinesterase–monoamine oxidase inhibitor–iron chelator radical scavenger drugs for Alzheimer’s disease with Parkinsonism, Parkinson’s disease with dementia, and Lewy body disease.^95^

For AD therapeutics we have introduced carbamate cholinesterase inhibitor (ChEI) moieties into HLA20 to give HLA20A (Figure 7) and into M30 to give M30C-N (Figure 8). And we have even added the glutamate antagonist, memantine, which is presently in clinical use (Figure 8). These compounds HLA20A and M30C-N have been shown to have potent ChE and MAO-A and B-inhibitory activities and possess similar neuroprotective activity to those of their parent compounds, HLA20 and M30.^32^

The accumulation of iron at sites where neurons degenerate in AD and PD is thought to be a major event that is linked to the neurodegenerative process.^41^ The novel non-toxic lipophilic (and therefore brain-permeable) iron chelator VK-28 and its multifunctional derivative, M30 (both of which possess the MAO-inhibitory and neuroprotective propargyl moiety of rasagiline), offer potential therapeutic benefits for PD. M30 at-tenuates apoptotic events in SH-SY5Y neuroblastoma cells in a serum deprivation model via multiple protection mechanisms, including 1) reduction of the proapoptotic proteins, Bad and Bax; 2) reduction of apoptosis-associated Ser139-phosphorylated H2A.X; 3) induction of the antiapoptotic protein, Bcl-2; and 4) inhibition of the cleavage and activation of caspase-3. M30 also promotes morphological changes, resulting in axonal growth-associated protein-43 (GAP-43), which is implicated in neuronal differentiation. The compound markedly reduces the levels of cellular holo-APP (amyloid precursor protein), the β-CTF (C-terminal fragment), and levels of amyloidogenic Aβ peptide in the medium of SH-SY5Y and CHO cells stably transfected with the APP “Swedish” mutation. In addition, levels of the non-amyloidogenic sAPPα in cell medium, as well as levels of α-CTF in cell lysate, were found to be elevated. These results are consistent with the presence of an iron-responsive element (IRE) in the 5′-untranslated region (5′UTR) of APP and demonstrate the effectiveness of M30 in limiting holo-APP expression and Aβ peptide secretion. Therefore, the multifunctional properties of M30 suggest that it may offer extraordinary potential as a drug for the treatment of PD, especially PD dementia^46^ and AD.^47^,^48^ More recently in a transgenic G93A superoxide dismutase (SOD) model of amyotrophic lateral sclerosis (ALS) this extends the life-span of these animals and has neurogenic activity in NCS-34 rat motor neurons by inducing neurite formation with increased neuronal GAP-43.^45^

## MONOAMINE OXIDASE INHIBITION BY A_2A_ RECEPTOR ANTAGONISTS

**Figure 9:**
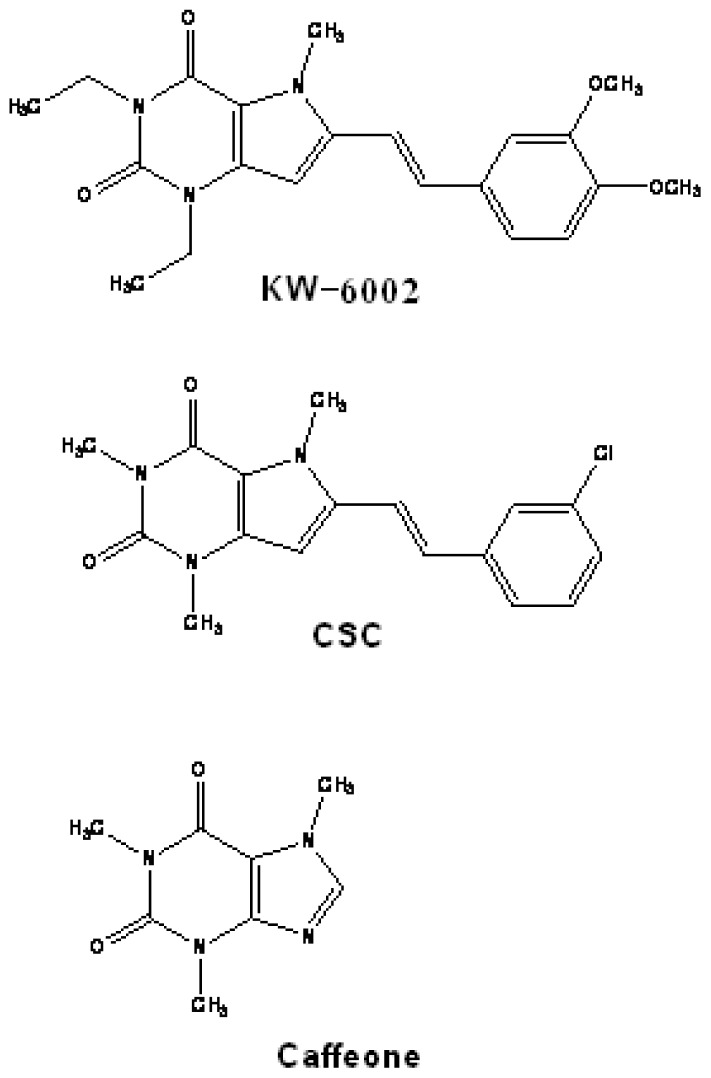
Structures of multimodal MAO-B and adenosine 2A receptor antagonists developed as anti-Parkinson drugs from caffeine.

**Figure 10:**
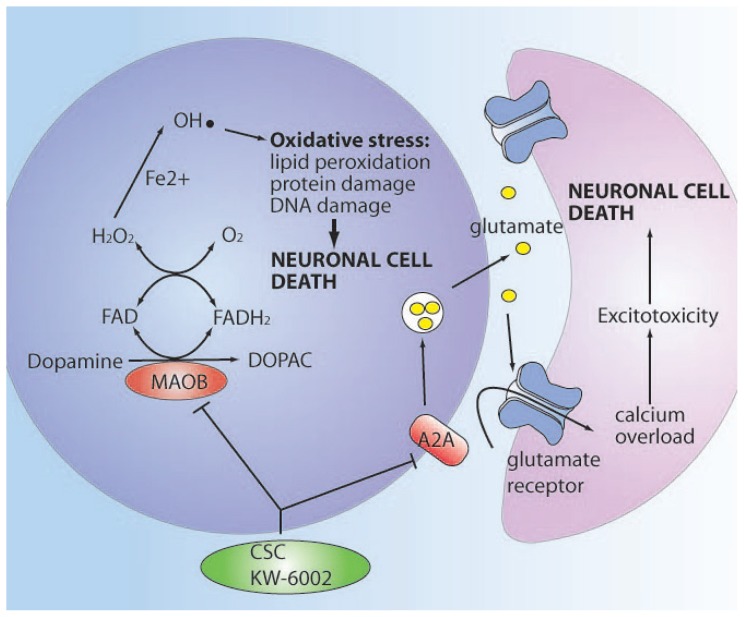
Dual molecular mechanism of the MAO-B/A_2A_ antagonists, CSC, and KW-6002, preventing neuron death by antioxidant effects via MAO-B inhibition and prevention of excitotoxic release of glutamate via A2_A_ inhibition.

In PD, a dual mechanism that includes inhibition of MAO-B, as well as adenosine A_2A_ receptor blockade, offers a novel therapeutic approach to prevent neuronal cell death (Figure 9, Figure 10). As detailed earlier, MAO-B plays a role in the catabolism of neurotransmitters such as DA, serotonin, and norepinephrine, leading to hydrogen peroxide formation which contributes to oxidative stress and neuronal cell death.^49^ Levels of MAO-B are found to be increased in older patients^50^–^52^ which has led to the rationale for the use of drugs such as selegiline (deprenyl) and lazabemide,^53^ and the design of drugs such as ladostigil^27^ as described before.

Caffeine, a non-selective adenosine receptor antagonist, is under some scrutiny as a potential drug to counteract age-related cognitive decline. Work in this regard is supported by evidence that critical changes in adenosine-related neurotransmission occur with aging and may be counteracted by adenosine receptor antagonists.^54^–^56^ Caffeine, in fact, has been suggested to protect against β-amyloid neurotoxicity,^55^ while acute treatment with caffeine and the A_2A_ receptor antagonist ZM241385 was recently found to reverse age-related olfactory deficits and memory decline in rats,^56^ clearly suggesting involvement of A_2A_, but not A^1^ receptors, in cognitive decline and possibly neurodegenerative processes. Evidence such as the preceding, and other evidence for neuroprotection also in Parkinsonian models, led Petzer et al.^57^ to evaluate (*E*)-8-styryl-xanthinyl-derived adenosine A_2A_ receptor antagonists for inhibition also of brain MAO-B. Included in these studies were KW-6002, a potent A_2A_ receptor antagonist (Ki of 2.2 nM) which is undergoing clinical trials for PD, and (*E*)-8-(3-chlorostyryl) caffeine (CSC), which has been shown to be neuroprotective in the MPTP Parkinsonian mouse model.^58^ All of the compounds tested in the studies by Petzer et al.^57^ showed MAO-B inhibition in the low micromolar to high nanomolar range, with the Ki of KW-6002 at 21 μM, and that of CSC at 0.1 μM. These results clearly suggest that the neuroprotective properties of KW-6002 and CSC may in part be due to MAO-B inhibition, in synergism with the A_2A_ antagonism (Figure 9).^59^

## NMDA (N-METHYL-D-ASPARTIC ACID) ANTAGONISM BY CALCIUM CHANNEL BLOCKERS

The divalent calcium cation plays an important role in neuronal cell death.^60^–^63^ One of the receptors activated by glutamate (together with its co-agonist glycine), the NMDA receptor, is a major conduit for the influx of calcium ions into cells under excitotoxic conditions. The prevention of such excessive influx of calcium (known as excitotoxicity) therefore remains a major drug target in the design of neuroprotective agents. Excess accumulation of calcium in neuronal cells rapidly leads to cell death through a variety of mechanisms including activation of proteases, nucleases, phospholipases, nitric oxide synthase (NOS), and other degradative enzymes that not only lead to activation of death cascades, but also to free radical formation.^63^ NMDA receptor antagonists such as dizocilpine (MK-801) and memantine may possess a dual mechanism by which neuronal cells are protected, both by direct blockade of the NMDA receptor and by attenuating tumor necrosis factor alpha (TNFα)-induced potentiation of glutamate toxicity.^64^

Brain injury after ischemic stroke also triggers a release of glutamate-associated excitotoxic events, and the incidence of cognitive impairment and dementia have both been reported to be elevated after cerebral stroke, especially in the elderly.^65^ Up to 25% of stroke patients exhibit symptoms of dementia, including symptoms reminiscent of PD dementia.^66^ Stroke is the third leading cause of death in the United States,^62^ and there is a definitive need to develop drugs that can protect or save neurons after an ischemic incident since, to date, no effective treatment has been developed to prevent neuronal cells from dying during stroke conditions.^60^

Several studies have shown that NMDA receptor antagonists, such as dizocilpine (MK-801) and the polycyclic cage amine memantine, display neuroprotective effects in experiments using ischemia paradigms in neurons.^60^,^67^–^69^ An alternative pathway for calcium to enter into neuronal cells is through voltage-gated ion channels, such as L-type calcium channels. Animal experiments with nimodipine have suggested that calcium channel antagonists may be neuroprotective in ischemia by antagonizing the influx of calcium into neuronal cells.^60^ The importance of calcium overload during cell death suggests that a dual calcium channel and NMDA receptor antagonist might be useful as a neuroprotective drug in stroke and other neurodegenerative disease such as idiopathic PD, where it has been suggested that brain-permeable L-type calcium channel blockers may have a salutary effect on the disease.

**Figure 11:**
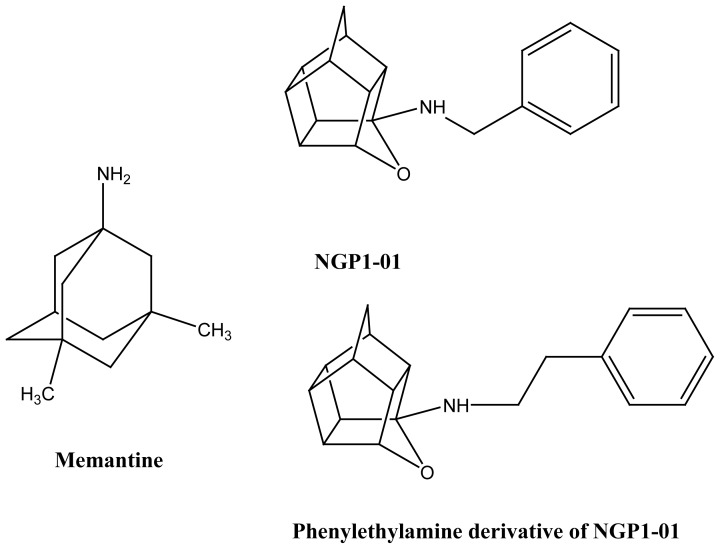
Structures of memantine-derived glutamate antagonists possessing calcium channel-blocking properties.

NGP1-01 (8-benzylamino-8,11-oxapentacyclo [5.4.0.0^2^,^6^.0^3^,^10^.0^5^,^9^]undecane) (Figure 11) is a polycyclic cage amine derived from the reductive amination of benzylamine and Cookson’s “bird cage” diketone, the biology of which was first described by Van der Schyf.^70^ The L-type calcium channel-blocking activity of NGP1-01 was investigated utilizing electrophysiological experiments in isolated guinea-pig papillary muscle and sheep Purkinje fibers.^70^ The structural similarity of NGP1-01 to another polycyclic cage amine and NMDA receptor antagonist, memantine, led to the evaluation of NGP1-01 for potential NMDA receptor antagonism. Memantine is an uncompetitive NMDA receptor antagonist which is used clinically to treat AD but has also been used for PD in Germany.^17^,^18^,^71^ Its favorable fast on-off binding kinetics gives this compound an improved side-effect profile compared with other N-methyl-D-aspartic acid (NMDA) antagonists such as MK-801.^71^ NGP1-01 was shown also to be an uncompetitive NMDA antagonist in murine whole brain synaptoneurosomes and blocked NMDA-mediated ^45^Ca^2+^ uptake with an IC_50_ of 2.98 μM.^72^

In a recent paper Kiewert et al.^73^ showed that NGP1-01 (at 1 μM) inhibited depolarization-induced calcium influx by 78% in cortical neurons preloaded with fura-2 AM, with a potency similar to that of nimodipine, while simultaneously inhibiting NMDA-induced (1 mM) calcium influx by 52%, only slightly less potent than memantine. Using *in-vivo* microdialysis, choline release was monitored during NMDA infusion as a measure of excitotoxic membrane break-down. Intraperitoneal injection of NGP1-01 (40 mg/kg) reduced NMDA-induced membrane break-down by 31% (*P* < 0.01) while memantine (10 mg/kg) (Figure 11) reduced choline release by 40%. These results demonstrate that NGP1-01 simultaneously blocks both major neuronal calcium channels and is brain-permeable after peripheral administration. This dual mechanism of modulating calcium entry into neuronal cells might suggest that NGP1-01 may have utility as a neuroprotective agent in PD, stroke, and other neurodegenerative diseases, especially in patients with co-morbidity among these diseases. This promise of neuroprotection has recently been partly confirmed with *in-vivo* studies using the middle cerebral artery occlusion (MCAO) mouse model of stroke, wherein it was shown that NGP1-01, administered 30 minutes before MCAO, provided substantial protection against cerebral ischemia-induced brain lesioning, as well as brain swelling measured 24 hours after MCAO.^74^

Another role assigned to cage amines such as NGP1-01 in PD therapy is the ability of these compounds to inhibit dopamine re-uptake into nerve terminals. Compounds that are able to block the dopamine transporter (DAT) have been suggested to be more useful in treating the motor symptoms in PD, as opposed to norepinephrine and serotonin re-uptake inhibitors.^75^ Additionally, compounds with the ability to block DAT may also have neuroprotective activity.^76^ NGP1-01 (Figure 11) was recently shown to block dopamine re-uptake in murine synaptosomes, with an IC_50_ of 57 μM. One of NGP1-01’s derivatives, a phenylethylamine derivative, was even more potent, with an IC_50_ of 23 μM.^77^ The latter compound was also found to be neuroprotective in the MPTP-Parkinsonian mouse model, affording protection against a single 35 mg/kg (i.p.) dose of 1-methyl-4-phenyl-1,2,3,6-tetrahydropyridine (MPTP).^78^

## GREEN TEA POLYPHENOLS

**Figure 12:**
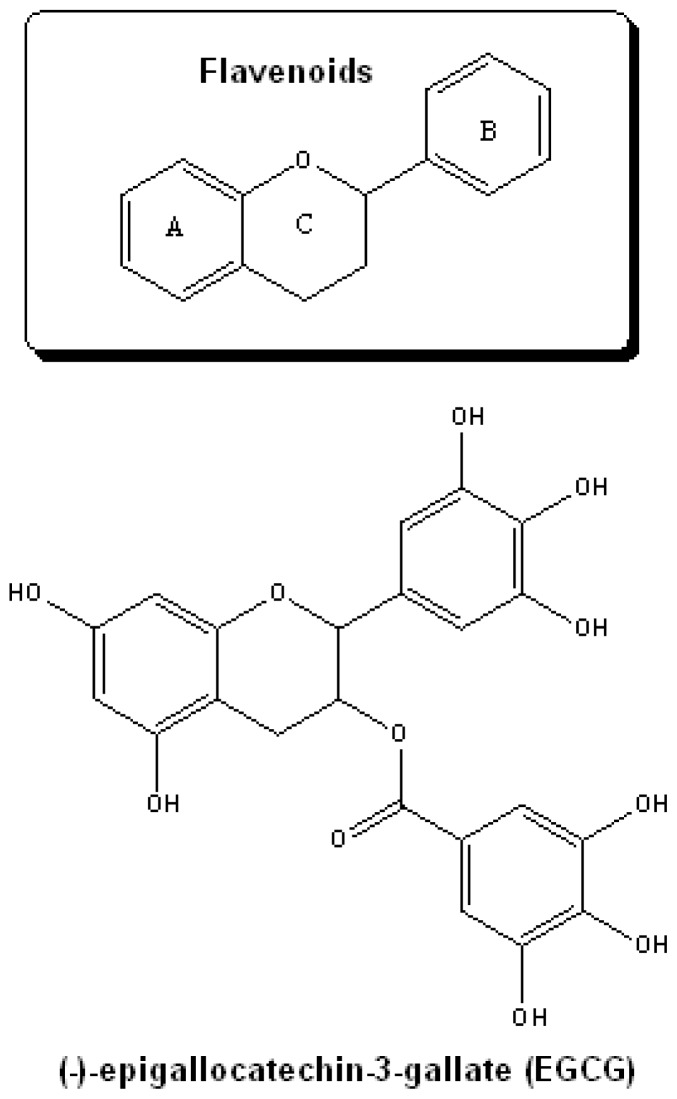
Structures of polyphenol flavenoids which in *in-vitro* and *in-vivo* studies have been shown to have neuroprotective and cognitive enhancing activities in animal models of Parkinson’s and Alzheimer’s diseases.

Polyphenols are natural products present in beverages such as red wine and tea.^79^ One of the classes of polyphenols that are pharmaceutically interesting is the flavenoids (Figure 12). These compounds are characterized by an aromatic ring which is condensed to a heterocyclic ring and attached to a second aromatic ring. An innovative therapeutic approach could be the use of natural plant polyphenol flavenoids, reported to have access to the brain and to possess multifunctional activities as iron chelators, radical scavengers, anti-inflammatory agents, and neuroprotectants.^80^–^83^

These compounds and their actions have been extensively reviewed.^84^ In particular, the major constituent of green tea catechin extract (-)-epigallocatechin-3-gallate (EGCG) (Figure 12) plays a major role in the prevention of neurodegeneration in a variety of cellular and animal models of neurodegenerative diseases.^85^ This effect appears to be mediated through multiple pathways, including the participation of the pro-survival PKC and extracellular mitogen-activated protein kinase (MAPK) signaling and the promotion of neurite outgrowth.^86^ Structurally important features defining their chelating potential are the 3′,4′-dihydroxyl group in the B ring,^80^ as well as the gallate group^87^ which may neutralize ferric iron to form redox-inactive iron, thereby protecting cells against oxidative damage.^88^ Recent studies have shown that prolonged administration of EGCG to mice induced a significant reduction in membrane-associated APP levels in hippocampus^89^ and in cerebral Aβ levels con-comitant with reduced β-amyloid plaques.^90^ This effect may be accounted for, in part, by the chelation of the intracellular free-iron labile pool, modulating APP mRNA translation via its IRE-type II,^91^ as has recently been described for other metal chelators, such as desferoxamine, clioquinol, and dimercaptopropanol.^92^,^93^

**Table:** Executive Summary: Multimodal Drugs Under Development

**Introduction** PD, AD, and ALS are progressive neurodegenerative diseases. Multiple molecular pathways are involved in the cell death process. Single targeted ligands may not modify the cell death process since the other pathways may still be contributing to cell death. Multifunctional ligands can be designed to target more than one drug target in PD, AD, and ALS.
**Rasagiline** Rasagiline is a second-generation MAO-B inhibitor. It is used for the treatment of PD. It has also been shown to be neuroprotective and neurorestorative. It may be the first neuroprotective disease-modifying drug.
**Ladostigil-Monoamine Oxidase-Cholinesterase** Ladostigil multifunctional drug derivative if rasagiline with a cholinesterase inhibitor moiety. For use in AD disease with Parkinsonism, Lewy body disease, and PD with dementia and depressive illness. It has been shown to have all the attributes of rasagiline as neuroprotective and neurorescue drug. It has antidepressant activity due to its MAO-A inhibitory activity.
**M30 and its Derivatives** Multimodal drug iron chelator–radical scavenger MAO-AB inhibitor, M30, M30C and M30P, HLA20, HLA20A. For use in PD, AD, and ALS. Neuroprotective and neurorestorative. Neurotrophic activity, BDNF, VEGF, erythropoietin. Inhibitor of cyclin D1 resulting in cell differentiation.
**Monoamine Oxidase/A** **_2A_** **Antagonists** Epidemiology has suggested caffeine use to be inversely related to PD. Dual MAO and A_2A_ antagonists have been shown to be neuroprotective.
**NMDA/L-Type Calcium Channel Blockers** Calcium overload in neuronal cells leads to cell death. Two major routes for calcium entry into cells are the NMDA receptor/ion channel as well as the L-type calcium channel. NGP1-01 is a dual-mechanism drug which blocks both the NMDA receptor/ion channel as well as the L-type calcium channel, with resulting neuroprotective activity shown *in vivo*. DAT inhibition by NGP1-01 may be additionally useful in treatment of the motor symptoms of PD.
**GREEN TEA CATECHINS** Multimodal green tea polyphenols, EGCG have neuroprotective properties. Multiple neuroprotective mechanisms are suggested including antioxidant and metal chelation.
**FUTURE PERSPECTIVE** Multifunctional drugs show great promise as neuroprotective-neurorescue agents in neurodegenerative diseases. Multiple drug targets can be targeted with a single compound, with less chance of side-effects such as associated with the practice of polypharmacy.

## CONCLUSIONS

PD and AD are complex diseases with multiple pathways which contribute to their etiology and finally cell death of DA, cholinergic, and other neurons. To address this multiplicity, compounds that have more than one target in the cell death cascades are now investigated and designed. These drugs have the advantage of acting at several sites in the brain and neurons and possess not only neuroprotective but also neurorestorative activity. Their neuroprotective activity relies on activating the Bcl-2 antiapoptotic proteins while down-regulating the proapoptotic proteins through gene regulation. On the other hand the neurorestorative property of these compounds is associated with induction of neurotrophins such as BDNF, GDNF, and HIF (hypoxia-inducing factor). The feasibility of moving these drugs to market has been shown through the success of rasagiline, which has been shown to have neuroprotective activity and has made it to the market as a PD therapeutic.^11^ The development of multimodal drugs is not limited to neurodegenerative disease; similar approaches are under way with other complex diseases such as cancer, AIDS, depressive illness, schizophrenia, and possibly cardiovascular disorders.^94^

## Abbreviations

5′UTR: 5′-untranslated region
AChE: acetylcholinesterase
AD: Alzheimer’s disease
AF: atrial fibrillation
ALS: amyotrophic lateral sclerosis
APP: amyloid precursor protein
BDNF: brain-derived neurotrophic factor
CSC: (*E*)-8-(3-chlorostyryl) caffeine
CTF: C-terminal fragment
DA: dopaminergic
DAT: dopamine transporter
EGCG: (-)-epigallocatechin-3-gallate
FAD: flavin adenine dinucleotide
FDA: Food and Drug Administration
GAP-43: growth-associated protein-43
GDNF: glia-derived neurotrophic factor
HIF: hypoxia-inducing factor
IC_50_: half maximal inhibitory concentration
IRE: iron-responsive element
M30: [5-(N-methyl-N-propargylaminomethyl)-8-hydroxyquinoline]
MAO: monoamine oxidase
MAPK: mitogen-activated protein kinase
MCAO: middle cerebral artery occlusion
MPTP: 1-methyl-4-phenyl-1,2,3,6-tetrahydropyridine
NGP1-01: (8-benzylamino-8,11-oxapentacyclo [5.4.0.0^2,6^.0^3,10^.0^5,9^] undecane)
NMDA: N-methyl-D-aspartic acid
PD: Parkinson’s disease
PKC: protein kinase C
sAPPα: soluble amyloid precursor protein alpha
SNpc: substantia nigra pars compacta
SOD: superoxide dismutase
TNF: tumor necrosis factor
VEGF: vascular endothelial growth factor.
