# Correction for the article “Why Do We Need Multifunctional Neuroprotective and Neurorestorative Drugs for Parkinson’s and Alzheimer’s Disorders?” by Moussa B. H. Youdim

**DOI:** 10.5041/RMMJ.10221

**Published:** 2015-10-26

**CORRECTION**

In the following paper: Youdim MBH. Why do we need multifunctional neuroprotective and neurorestorative drugs for Parkinson’s and Alzheimer’s disorders? *Rambam Maimonides Med J* 2010;1(2):e0011. doi:10.5041/RMMJ.10011, it was noted that the Acknowledgement regarding the source of the text was not published. The following Acknowledgement has been added:

**Acknowledgement:** This article is reprinted with slight modifications from Youdim MBH. Why Do We Need Multifunctional Neuroprotective and Neurorestorative Drugs for Parkinson’s and Alzheimer’s Diseases as Disease Modifying Agents. Exp Neurobiol. 2010;19(1):1–14, under the terms of the Creative Commons Attribution License (http://creativecommons.org/licenses/by-nc/3.0/).

The HTML and PDF versions of the paper have been corrected in the RMMJ.org.il archives.

Published: August 31, 2015

